# New Directions in the Investigation of Loneliness and Social Connection Across Time Scales and Contexts

**DOI:** 10.1093/geroni/igaf122.1681

**Published:** 2025-12-31

**Authors:** Karina Van Bogart, Eileen Graham, Susan Charles

## Abstract

This symposium will focus on novel methodology and approaches to investigating loneliness and social connection across the lifespan and influences on health and aging. Although loneliness has been shown to be a risk factor for poor health outcomes and greater social connection has been linked to better health and well-being, more research is needed on nuances underlying these associations. In keeping with the GSA program theme this year (Innovative Horizons in Gerontology), such work is needed to push the field forward and help elucidate temporal and contextual factors that may be important targets for future interventions that aim to promote social connection and offset related negative health outcomes. This symposium will begin with a brief introduction to the value of the overall topic (Graham), followed by four empirical talks that will highlight different approaches to better understanding social connection in later life. The first talk will present a coordinated analysis examining the link between loneliness and social isolation with cognitive health span across 11 datasets (Yoneda). A second talk will present a coordinated analysis examining bivariate change in social asymmetry (discordance between loneliness and social isolation) and cognitive function across midlife and older adulthood (Van Bogart). The third talk will focus on how trait-like loneliness relates to fluctuations in loneliness and social threat detection in daily life (Shao and Ong). The final talk will focus on a novel conceptualization of social connection dynamics in daily life (Rush). The discussant (Susan Charles) will highlight the importance and innovation of this work.

